# Supplementary material for: Analysis of Gastric Cancer Transcriptome Allows the Identification of Histotype Specific Molecular Signatures With Prognostic Potential
Source: Front Oncol. 2021 May 3;11:663771. doi: 10.3389/fonc.2021.663771 (PMC8126708; doi:10.3389/fonc.2021.663771)
Supplement: Supplementary file 1 [file Table_1.docx]

**Supplementary Table 1. Principal pathways of Proliferation, differentiation and metabolism Cluster for the subset AB.**

| Proliferation, Differentiation and Metabolism | Up-regulated genes | Down-regulated genes |
| --- | --- | --- |
| Cell Cycle | PLK1, PCNA, ESPL1, MCM2, CDC25B, CCNA2, CDC45, PTTG1, CDK1, PKMYT1, CDC20, CCNE1, CDC25C, SFN, CDC6, MCM3, MCM4, ORC1, ORC6, BUB1, CCNB2, CCNB1, E2F5, E2F2, E2F3, CDC25A, TTK | CDKN1C |
| Mitotic G2-G2/M phases | CDC25C, MYBL2, TPX2, PKMYT1, AJUBA, CCNB1, CDC25B, CDC25A, PLK1, BORA, FOXM1, CCNB2, CDK1, AURKA, CENPF, GTSE1, HMMR |  |
| Ectoderm Differentiation | TNFRSF11B, HIST1H2BH, RHPN1, LY6E, TFAP2A | PLCXD3, FZD4, LDB2, ARHGDIG, PRKAG2, ARHGAP15, GLI3, ZBTB16, PTPN13, ARHGAP10 |
| Mitotic G1-G1/S phases | MCM10, TOP2A, CDC25A, CDC45, CCNE1, CCNA2, RRM2, CDK1, MYBL2, DHFR, CDC6, ORC1, TK1, TYMS, PCNA |  |
| Senescence and Autophagy in Cancer | PLAU, CDC25B, CXCL1, IL8, PCNA, IL1A, IL1B, CCL3, TNFSF15, INHBA, HMGA1 | IGF1, BCL2, GABARAPL1 |
| Epithelial to mesenchymal transition (EMT) | GDF15, TMPRSS4, CLDN1, COL4A1, FOXM1, CLDN3, CLDN4, CLDN7, WNT5A | EIF5A2, COL4A3, COL4A5, WNT2B, FZD4 |
| Focal Adhesion | ITGA2, SPP1, LAMC2, COL4A1 | LAMA2, PDGFD, CHAD, BCL2, ITGA8, COL2A1, IGF1, TNXB, MAPK10 |
| Circadian rhythm related genes | TYMS, CLDN4, TNFRSF11A, TOP2A | ADA, SLC9A3, NTRK3, ID4, PRKAA2, PTGDS, RORB, MAPK10 |
| G1 to S cell cycle control | E2F2, PCNA, CDK1, CCNE1, CCNB1, MCM4, MCM3, MCM2, E2F3, CDC25A | CDKN1C |
| Metapathway biotransformation Phase I and II | HS3ST1 | CYP4B1, GSTA1, GLYATL1, GPX3, CYP1B1, SULT2A1, GSTM2, GSTM5, FMO2 |
| Adipogenesis | LIF, HMGA1, OSM, AGPAT2 | IGF1, PTGIS, SLC2A4, LIFR, RXRG, KLF15 |
| DNA Damage Response | PMAIP1, FANCD2, CDC25A, SFN, CCNB1, CDC25C, CCNE1, CCNB2, RAD51 |  |
| Regulation of Actin Cytoskeleton | VIL1, F2R | CHRM3, FGF14, PIK3C2G, FGF2, CYFIP2 |
| DNA Replication | RFC4, PCNA, MCM2, MCM10, MCM4, CDC6, MCM3 |  |
| ESC Pluripotency Pathways | WNT5A, LIF | FZD4, LIFR, FGF14, FGF2, WNT2B |
| Mesodermal Commitment Pathway | INHBA, WDHD1, NCAPG2 | PBX3, SLC2A12, TOX, FZD4 |
| Endoderm Differentiation | WDHD1, NCAPG2 | SFRP1, TCEAL2, PBX3, SLC2A12, TOX |
| Differentiation Pathway | INHBA, IL11, WNT5A | FGF2, KIT, IGF1, WNT2B |
| Cell Cycle Checkpoints | BUB1B, CDC20, CDC25A, CDC25C, PKMYT1, CLSPN, MAD2L1 |  |
| Prostaglandin Synthesis and Regulation | S100A10, ANXA2S, OX9 | PTGIS, PTGDS, PTGER3 |
| Glycolysis and Gluconeogenesis | PFKP, LDHA, ENO1, HK2 | SLC2A4, FBP2 |
| Synthesis of DNA | MCM3, CDC45, FEN1, CDC6, GINS1, MCM2 |  |
| Integrin-mediated Cell Adhesion | ITGA2 | SEPP1, CAPN6, ITGA8, ITGAL, MAPK10 |
| Trans-sulfuration pathway | LDHA | CKM, CDO1, CKB, CKMT2 |
| M/G1 Transition | ORC1, MCM10, CDC6, CDC45, ORC6 |  |
| Apoptosis | PMAIP1, BIRC5 | MAPK10, IGF1, BCL2 |
| Matrix Metalloproteinases | MMP12, MMP10, MMP3, MMP1 | TIMP3 |
| Oxidative Damage | PCNA | CDKN1C, BCL2, MAPK10 |
