# Supplementary material for: Analysis of Gastric Cancer Transcriptome Allows the Identification of Histotype Specific Molecular Signatures With Prognostic Potential
Source: Front Oncol. 2021 May 3;11:663771. doi: 10.3389/fonc.2021.663771 (PMC8126708; doi:10.3389/fonc.2021.663771)
Supplement: Supplementary file 2 [file Table_2.docx]

**Supplementary Table 2. Principal pathways of Inflammation Cluster for the subset AB.**

| Inflammation | Up-regulated genes | Down-regulated genes |
| --- | --- | --- |
| IL-18 signaling pathway | CCL3, MMP3, CLDN1, PLA2G7, IL1B, CCL20, CXCL16, MMP1, LMNB2, ENO1 | BCL2, SYT10, CD36 |
| Chemokine signaling pathway | CXCL3, CCL20, CCL3, CCL15, CXCL5, CXCL16, LYN | CCL21, CXCL12, GNG7, ADCY2, DOCK2 |
| Spinal Cord Injury | IL1B, MMP12, CXCL1, IL1A, CXCL2, E2F5, COL4A1, SOX9, CDK1 | AQP4, COL2A1 |
| Breast cancer pathway | RAD51, E2F2, E2F3, WNT5A | IGF1, KIT, PGR, FGF2, FZD4, WNT2B |
| Complement and Coagulation Cascades | F2R, PLAU, PLAUR | F10, C7 |
| Cytokines and Inflammatory Response | IL1A, IL1B, CXCL2, CXCL1, IL11 |  |
| Signal transduction through IL1R | IRAK2, IL1RN, IL1A, IL1B |  |
| Cells involved in local acute inflammatory response | IL1A, IL8 | C7, ITGAL |
| LTF danger signal response pathway | IL1A, IL1B, IL8 | LTF |
| Interleukin-6 family signaling | IL11, LIF, OSM | LIFR |
| FasL pathway and Stress induction of HSP regulation | IL1A, LMNB1, LMNB2 | BCL2 |
| Interleukin-11 Signaling Pathway | IL11, BIRC5, ITGA2 | BCL2 |
