# Supplementary material for: Analysis of Gastric Cancer Transcriptome Allows the Identification of Histotype Specific Molecular Signatures With Prognostic Potential
Source: Front Oncol. 2021 May 3;11:663771. doi: 10.3389/fonc.2021.663771 (PMC8126708; doi:10.3389/fonc.2021.663771)
Supplement: Supplementary file 3 [file Table_3.docx]

**Supplementary Table 3. Principal pathways of Signaling Cluster for the subset AB.**

| Signaling | Up-regulated genes | Down-regulated genes |
| --- | --- | --- |
| PI3K-Akt signaling Pathway | CCNE1, LAMC2, ITGA2, COL4A1, SPP1, ANGPT2, EFNA3, EPHA2, OSM, F2R, PIK3AP1 | CHAD, COL4A5, ITGA8, COL2A1, TNXB, LAMA2, COL4A3, GHR, ANGPT1, FGF14, FGF2, IGF1, PDGFD, KIT, LPAR1, GNG7, PRKAA2, PPP2R3A, BCL2 |
| Focal Adhesion-PI3K-Akt-mTOR-signaling pathway | LAMC2, ITGA2, COL4A1, SPP1, ANGPT2, EFNA3, EPHA2, OSM, F2R | CHAD, ITGA8, COL2A1, TNXB, LAMA2, ITGAL, GHR, ANGPT1, FGF14, FGF2, IGF1, PDGFD, KIT, LPAR1, GNG7, CAB39L, PRKAA2, PPP2R3A, SLC2A4, HIF3A |
| Nuclear Receptors Meta-Pathway | SLC7A11, EPHA2, CCL20, IL11, IL1B, MYOF, ENC1, TNS4, CDK1, SLC6A14 | SDPR, PDK4, TSC22D3, GPX3, SULT2A1, GSTA1, CYP1B1, SLC2A4, CDKN1C, PMP2, SLC2A12, DNER, GSTM5, GSTM2, SLC5A5 |
| Vitamin D Receptor Pathway | MXD1, KLK6, SPP1, S100A2, CD9, TNFRSF11B, CST1, CCNE1 | TRPV6, SULT2A1, ID4, SLC2A4, TIMP3, DNER, SFRP1, BMP6 |
| NRF2 pathway | SLC7A11, EPHA2, SLC6A14 | GSTA1, GPX3, SLC2A12, SLC2A4, GSTM5, GSTM2, SLC5A5 |
| Gastrin Signaling Pathway | BIRC5, ANXA2, IL8, CLDN1 | CHGA, HDC, CCKBR, SLC9A3, KIT |
| Wnt Signaling | PLAU, WNT5A, FOSL1 | WNT2B, FZD4, CAMK2B, MAPK10, SFRP1, PRICKLE2 |
| VEGFA-VEGFR2 Signaling Pathway | PLAU, SHB, TEAD4, MMP10, PLAUR | ITPR1, RKAA2, BCL2, TNXB |
| Glucocorticoid Receptor Pathway | IL11, ENC1, CCL20, TNS4 | PMP2, TSC22D3, CDKN1C, DNER, SDPR |
| MAPK Signaling Pathway | IL1A, CDC25B, IL1B | MAPK10, CACNA1A, CACNA2D2, FGF2, FGF14 |
| Regulation of toll-like receptor signaling pathway | CD80, IRAK2, SPP1, CCL3, PLK1, IL1B, IL8 | MAPK10 |
| GPCRs, Class A Rhodopsin-like | F2R | CHRM3, DRD5, HTR1E, CCKAR, CCKBR, PTGER3 |
| Wnt Signaling Pathway and Pluripotency | PLAU, WNT5A, FOSL1 | WNT2B, MAPK10, FZD4, PPP2R3A |
| PPAR signaling pathway | SLC27A2, MMP1 | RXRG, FABP3, CD36, FABP4, ACADL |
| G-Protein Signaling Pathways |  | GNG7, GNAZ, GNAO1, ADCY2, ITPR1, PRKAR2B, PDE1A |
| JAK/STAT | IL1RN, IL1B, AURKA | GHR, LEPR, IGF1, PRKAA2 |
| Toll-like Receptor Signaling Pathway | CD80, SPP1, CCL3, IL1B, IL8 | MAPK10 |
| Ras Signaling | EPHA2, CALML4, ETS2 | KIT, GNG7, MAPK10 |
| TGF-beta Signaling Pathway | CCNB2, CDK1, E2F5, MMP12, MMP1, ITGA2 |  |
| ErbB Signaling Pathway | EREG | ERBB4, CAMK2B, MAPK10 |
| TGF-beta Receptor Signaling | SPP1, LIF, INHBA | ZNF423 |
| Insulin Signaling |  | PIK3C2G, MAPK10, SLC2A4, PRKAA2 |
| Regulatory circuits of the STAT3 signaling pathway | F2R | GHR, LIFR, MAPK10 |
| EGF/EGFR Signaling Pathway | AURKA, MYBL2, PCNA | SH3GL2 |
| Regulation of mitotic cell cycle | NEK2, PTTG1, CDC20, BUB1B |  |
| Transcriptional regulation by the TAFP2 | MYBL2, ATAD2, NOP2 | KIT |
| Leptin signaling pathway | IL1RN, IL1B | LEPR, PRKAA2 |
| Regulation of DNA replication | CDC6, ORC1, MCM3, MCM2 |  |
| Cell surface interactions at the vascular wall | EPCAM, ANGPT2 | ANGPT1, JAM2 |
| GPCRs, Other | F2R | GPR133, CHRM3, CCKBR |
