# Supplementary material for: Analysis of Gastric Cancer Transcriptome Allows the Identification of Histotype Specific Molecular Signatures With Prognostic Potential
Source: Front Oncol. 2021 May 3;11:663771. doi: 10.3389/fonc.2021.663771 (PMC8126708; doi:10.3389/fonc.2021.663771)
Supplement: Supplementary file 4 [file Table_4.docx]

**Supplementary Table 4. Principal pathways of Inflammation Cluster for the subset A.**

| Inflammation | Up-regulated genes | Down-regulated genes |
| --- | --- | --- |
| IL-18 signaling pathway | IRAK1, IL18, IFNG, TOMM40, HMOX1, PYGB, ZC3H12A | CCL2, TRAF1, ACACB, TF, CA11 |
| Spinal Cord Injury | IFNG, MMP9, ANXA1, NOX4 | CCL2, PLA2G6, XYLT1 |
| IL1 and megakaryocytes in obesity | MMP9, IRAK1, IL18, IFNG | CCL2 |
| Type II interferon signaling (IFNG) | IFNG, PSMB9, OAS1 |  |
| Interleukin-6 family signaling | CLCF1 | CRLF1, CNTFR |
| Chemokine signaling pathway | CCL22, CCL28 | SHC2 |
| Development and heterogeneity of the ILC family | IFNG, IL18, AREG |  |
| TNF alpha Signaling Pathway | NOXO1 | TRAF1, CCL2 |
| TNF related weak inducer of apoptosis Signaling Pathway | MMP9 | TRAF1, CCL2 |
| Prostaglandin Synthesis and Regulation | ANXA4, ANXA1 |  |
| Toll-like Receptor Signaling Pathway | IRAK1 | IFNA16 |
