# Supplementary material for: Analysis of Gastric Cancer Transcriptome Allows the Identification of Histotype Specific Molecular Signatures With Prognostic Potential
Source: Front Oncol. 2021 May 3;11:663771. doi: 10.3389/fonc.2021.663771 (PMC8126708; doi:10.3389/fonc.2021.663771)
Supplement: Supplementary file 5 [file Table_5.docx]

**Supplementary Table 5. Principal pathways of Signaling Cluster for the subset A.**

| Signaling | Up-regulated genes | Down-regulated genes |
| --- | --- | --- |
| VEGFA-VEGFR2 Signaling Pathway | SPHK1, ANXA1, RND1, F3 | ACACB, GAB1, SHC2, PRKCE, CCL2, ADAMTS1 |
| Nuclear Receptors Meta-Pathway | IFNG, FASN, GCLM, HMOX1, SLC6A20 | CCL2, FKBP5, GSTA4 |
| Histone Modifications | HIST1H3D, HIST1H3J, HIST1H3C, HIST1H3G, HIST1H4L | PRDM2 |
| Insulin Signaling | TRIB3, GRB14 | SHC2, GAB1 |
| Ras Signaling | RIN1 | SHC2, GAB1, PLA2G6 |
| PI3K-Akt Signaling Pathway | LAMB3, CREB3L1 | IFNA16, COL9A3 |
| NRF2 pathway | GCLM, HMOX1, SLC6A20 | GSTA4 |
| Brain-Derived Neurotrophic Factor (BDNF) signaling pathway | DPYSL2 | SHC2, ACACB, GRIA3 |
| ErbB Signaling Pathway | AREG | GAB1, SHC2 |
| Statin Pathway | APOC2, APOC1, APOE |  |
| G Protein Signaling Pathways |  | PDE1C, PDE8B, PRKCE |
| Vitamin D Receptor Pathway | SLC34A2, SLC37A2 | ADRB2 |
