# Supplementary material for: Analysis of Gastric Cancer Transcriptome Allows the Identification of Histotype Specific Molecular Signatures With Prognostic Potential
Source: Front Oncol. 2021 May 3;11:663771. doi: 10.3389/fonc.2021.663771 (PMC8126708; doi:10.3389/fonc.2021.663771)
Supplement: Supplementary file 6 [file Table_6.docx]

**Supplementary Table 6. Principal pathways of Proliferation, differentiation and metabolism Cluster for the subset B.**

| Proliferation, Differentiation and Metabolism | Up-regulated genes | Down-regulated genes |
| --- | --- | --- |
| Focal Adhesion | LAMA5, MYLK, COL4A2, FN1, PGF, ITGA3, PDGFB | PAK3, FIGF, BLK, AKT3, PRKCB, PARVG, VAV3 |
| Class A/1 (Rhodopsin-like receptors) | HCAR3, HCAR2, CXCR1, FPR1, FPR2, CXCR2, CCRL2 | PTGER1, PTGFR, CCL19 |
| Mesodermal Commitment Pathway | EXT1, SNAI1, BHLHE40, C1QBP | SESN1, CSRP2, BMP4, VAV3, SOX21, TET1 |
| Endoderm Differentiation | C1QBP, NME1, EXT1, EZH2 | SOX21, FOXN3, OTX2, TET1, SESN1, VAV3 |
| Regulation of Actin Cytoskeleton | FGFR4, MYLK, FN1, PDGFB | FGF10, MRAS, FGF7, PAK3 |
| Genotoxicity pathway | PLK3,E2F7,HIST1H2BN,IKBIP,DUSP14 | ARRDC4, ACTA2, TNFRSF17 |
| Epithelial to mesenchymal transition in colorectal cancer | SNAI1, FN1, EZH2, COL4A2 | AKT3, SNAI2, ZEB2, CLDN11 |
| Senescence and Autophagy in Cancer | E2F1, SERPINE1, FN1, CXCR2, SLC39A4 | CXCL14, IGFBP5 |
| Cholesterol metabolism | DHCR7, EBP, SQLE, ELOVL2, FADS2, SCD | HMGCS2 |
| ESC Pluripotency Pathways | PDGFB, FGFR4 | AKT3, FGF10, BMPR1B, FGF7, BMP4 |
| Ectoderm Differentiation | PODXL, ELOVL2 | DMD, CLDN11, RGMA, NR2F2, BMP4 |
| Adipogenesis | SERPINE1, SCD, E2F1, SOCS3 | BMP4, NR2F1, PRLR |
| Glycolysis and Gluconeogenesis | ALDOA, GAPDH, PGK1, SLC2A1, PGAM1 | ALDOB |
| Pyrimidine metabolism | CDA, TYMP, RRM1, NME1, PNP, POLR2H |  |
| Apoptosis-related network due to altered Notch3 | RIPK2, HELLS, CARD14, SOCS3 | VIM, VAV3 |
| Apoptosis Modulation and Signaling | IL1R2, BAG3, BID | BLK, PRKD1, BMF |
| DNA Damage Response | BID, E2F1, CCNB3, H2AFX | SESN1 |
| Tryptophan metabolism |  | ACAT1, INMT, ALDH1A1, HAAO, TPH1 |
| Oxidation by Cytochrome P450 | CYP2B6, CYP2D6, CYP4F3 | CYP4X1, CYP4Z1 |
| Glycolysis Pathway D (2) | ALDOA, GAPDH, PGK1, PGAM1 | ALDOB |
| Oxidative Stress | SOD2, XDH | NFIX, SOD3, MAOA |
| Differentiation Pathway | CXCR1, PDGFB | BMP4, FGF10, FLT3LG |
| TGF-B Signaling in Thyroid Cells for Epithelial-Mesenchymal Transition | FN1, SNAI1 | VIM, SNAI2 |
| Oncostatin M Signaling Pathway | SOCS3, OSMR, SERPINE1 | PRKCB |
| Fatty Acid Omega Oxidation | CYP2D6 | ADH1C, ALDH1A1, ADH7 |
| Integrin-mediated Cell Adhesion | ITGA3 | VAV3, PAK3, AKT3 |
| Folate Metabolism | SLC19A1, SOD2 | FOLR4, SOD3 |
| Eicosanoid metabolism via Lipo Oxygenases (LOX) | FPR2 | HPGD, LTC4S |
| Eicosanoid metabolism via Cyclo Oxygenases (COX) | PTGS2 | HPGD, PTGFR |
